# Post Mortem Appearances in a Recent Dislocation of the Femur and Fracture of the Cranium

**Published:** 1848-08

**Authors:** 


*Post Mortem appearances in a recent Dislocation of the Femur and Fracture of the Cranium.* Reported by Erasmus D. Fenner, M. D., of New Orleans.

As an opportunity to examine the state of the parts in so important a luxation as that of the femur, is of rare occurrence, the following statement may not be uninterresting to the profession. Luxations of the extremities never proving fatal of themselves, the surgeon may meet and contend with them through a long life, without being able to know precisely the nature and amount of injury that is done. This is particularly the case with the *hip joint,* which is so strongly knit, that it can only be displaced by extraordinary force; and when dislocated, can only be replaced by great surgical skill and adroitness. It is generally believed, that the chief difficulty consists in overcoming the spasmodic contraction of the powerful muscles which lie around the hip joint; whilst it has been barely surmised, that the *capsular ligament* itself might present no inconsiderable obstacle to the return of the head of the femur to its proper place, after sufficient extension had been made. Mr. Samuel Cooper, in his Surgical Dictionary, quotes as follows, from Mr. Pott:

"The mere bones composing the articulations, or the mere connecting ligaments, would, in general, afford very little opposition; and the replacing the dislocation, would require very little trouble or force, were it not for the resistance of the muscles and tendons attached to, and connected with them: for, by examining the fresh joints of the human body, we shall find, that they not only are all moved by muscles and tendons, but also, that, although what are called the ligaments of the joints do really connect and hold them together in such manner as could not well be executed without them, yet in many instances they are, when stripped of all connexion, so very weak and lax, and so dilatable and distractile, that they do little more than connect the bones and retain the synovia; and that the strength as well as the motion of the joints depends in a great measure on the muscles and tendons connected with and passing over them; and this in some articulations which are designed for the greatest quantity, as well as for celerity of motion. Hence, it must follow, that as the figure, mobility, action and strength of the principal joints depend so much more on the muscles and tendons in connection with them, than on their mere ligaments, the former are the parts which require our first regard, those being the parts which will necessarily oppose us in our attempts for reduction, and whose resistance must be either eluded or overcome—terms of very different import, and which every practitioner ought to be well apprized of."

Mr. Cooper says: "That the muscles are the chief cause of resistance, is strongly evidenced by cases in which the dislocation is accompanied with injury of a vital organ; for then the bone may be reduced by a very slight force. Thus, in a man who had an injury of his jejunum, and dislocation of his hip, the bone was most easily replaced. (*Sir A. Cooper's Surgical Essays, part* 1, *p.* 20.) In short, anything which produces faintness or weakness, facilitates the reduction—as intoxication, nausea and sickness, paralysis, &c."

Again, "The supposition of the reduction being sometimes prevented by the capsular ligaments, Sir A. Cooper considers erroneous; he assures us, that in dislocations from violence, those ligaments are always extensively lacerated; and that the idea of the neck of the bone being girt or confined by them, is altogether untrue."

Now let us see what we may learn from our case.

*Case.*—H. B., an athletic fireman on one of the tow-boats, (Porpoise) plying between this city and the mouth of the river, was blown up and terribly injured by the bursting of the boilers, on the morning of the 14th June, 1847. The boat was descending the river, having a ship and brig in tow. All the boilers bursted at once, killing, wounding, and scalding a number of persons. The subject of our case was badly scalded and received a dreadful injury on the head. He was brought to the Charity Hospital on the 16th, and admitted into surgical ward No. 3, under the care of Dr. W. T. Brent. When he entered, he appeared to be laboring under a raving delirium, singing and talking incessantly. His condition was considered altogether hopeless, and scarcely anything was attempted to be done for him. He died on the night of the 17th, more than three days after the receipt of injury, and the autopsy was made on the morning of the 18th.

*Post-mortem appearances.—Head.*—There was found a fracture extending across the whole frontal portion of the head, involving the frontal, the temporal, the ethmoid, the malar, the superior maxillary, the nasal, and the sphenoid bones. The *crista galli* was broken off and driven through the dura mater, lacerating it considerably. There was considerable depression of the bones across the obitar region, and the brain was badly lacerated and bruised. The integuments at this point were *not cut or lacerated* but scalded and swollen. He was in the act of putting a stick of wood into the fire when the boiler bursted, and drove it forcibly against his head. It is worthy of remark, that the brain, where the phrenologists have located the organs of *language and tune*, suffered the greatest injury in this case, and that the man was *talking and singing* as long as he was able. The examination of the brain was not carried any farther, as our special attention was invited to the dislocated hip. As before remarked, no effort had been made to reduce the dislocation, the case being considered utterly hopeless.

*State of the dislocated Thigh.*—The luxation was upwards on the *dorsum ilii* of the left side; the limb was shortened about an inch and a half, and the toes were turned inwards. On raising the integuments, extensive ecchymosis was discovered around the joint, along the fascia superficialis, and amongst the muscles as low as the knee. On raising the gluteus magnus and medius, the naked head of the femur was exposed, lying on the dorsum of the ilium, with the *ligamentum teres* hanging to it. This ligament had been completely detached from the acetabulum, and nearly half of its attachment to the head of the femur was broken up. The head of the femur, in being forced upwards and outwards, had ruptured and lacerated a portion of the obturator externus, pyriformis, and gemini muscles. After all the external muscles about the joint had been dissected up, we could not bring down the thigh. The iliacus and psoas magnus were now severed. These let the head down somewhat, but it could not be replaced. The triceps adductor was now divided, but without effect. The ileo-femoral ligament was found tensely stretched. All the muscles between the pelvis and thigh being now severed, there was of course no obstacle to complete extension; yet it was found *impossible to replace the head of the femur into its proper position. It could not be forced back through the aperture in the capsular ligament out of which it had passed.* The capsular ligament was found ruptured at least one-half of its extent, yet the opening *had to be enlarged from one-half to three-quarters of an inch, before the head of the femur could be put back into the cotyloid cavity.* So, here evidently laid the chief obstacle to the reduction of the dislocation.

The *capsular* is a very strong fibrous ligament, and we may readily conceive of its possessing sufficient *elasticity* to allow the smooth round head of the femur to pass out through a lacerated opening, which might at once contract so as to offer considerable resistance to its return.

In Sir Astley Cooper's Essays on Dislocations and Fractures of the joints, may be found a case and examination similar to this, in which the man died twenty-four hours after the receipt of injury, but the dislocation was reduced before death. With due deference to the high authority of Sir Astley Cooper, I cannot help supposing the capsular ligament may often cause much of the difficulty in reducing dislocations of the hip joint. —*New Orleans Journal.*

